# Large inferior retinectomies for proliferative vitreoretinopathy in silicone oil-filled eyes

**DOI:** 10.1186/s40942-022-00420-1

**Published:** 2022-10-01

**Authors:** Gabriel Castilho Sandoval Barbosa, Allan Gomes da Silva, Guilherme Daher Gonçalves Monteiro dos Reis, Frederico Hackbart Bermudes, Carolina Maria Barbosa Lemos, Rafael Garcia, Thiago José Muniz Machado Mazzeo, Cleide Guimarães Machado, André Marcelo Vieira Gomes

**Affiliations:** 1 Department of Ophthalmology, Suel Abujamra Institute, São Paulo, Brazil; 2 Department of Ophthalmology, University of São Paulo, São Paulo, Brazil

**Keywords:** Retinal detachment, Proliferative vitreoretinopathy, Silicone oil, Retinectomy

## Abstract

**Background:**

To describe the anatomical and functional outcomes and late complications in patients who developed inferior proliferative vitreoretinopathy (PVR) in silicone oil-filled eyes and who required reoperation with large inferior retinectomy.

**Methods:**

This is a single-center, retrospective, interventional case series analysis. The study involved 18 individuals with tractional retinal re-detachment due to PVR development inferiorly in eyes who had undergone prior pars plana vitrectomy and silicone oil as a tamponade. All patients included in the study underwent secondary surgery with large inferior retinectomy (from 120° to 270°) and silicone oil filling.

**Results:**

The mean follow-up period was 44.0 ± 31.5 (± SD) months (range: 4 to 96 months. The anatomical success, defined as the complete reattachment of the retina until the last follow-up, was observed in 88.9% of the cases. The postoperative visual acuity ranged from 20/100 to hand motion at 60 cm. Only two cases (11.1%) did not achieve anatomical success at the last follow-up due to recurrent PVR and retinal re-detachment (one including hypotony). All of the patients were pseudophakic. The PVR grade, as well as the presence of PVR prior to primary surgery, showed no statistical correlation with BCVA, the extent of retinectomies, and final macular status. There was a statistically significant correlation between "Final BCVA" and "Initial BCVA" (r = 0.654) and between "Final BCVA" and "Extent of Retinectomy" (r = 0.615).

**Conclusions:**

Reoperation in eyes filled with silicone oil may be required when PVR is developed. Secondary surgery in these cases with large inferior retinectomy and silicone oil implantation may reach good anatomical success with low rates of late complications, besides improving visual acuity. A better BCVA at the time of re-RD diagnosis and cases of retinectomies with greater extensions showed a positive correlation with better functional outcomes.

*Trial registration* Research Ethics Committee of the Suel Abujamra Institute reviewed and approved this study protocol (approval number, 5.404.961).

**Supplementary Information:**

The online version contains supplementary material available at 10.1186/s40942-022-00420-1.

## Introduction

The term Proliferative Vitreoretinopathy (PVR) was proposed and introduced as a unifying definition and staging by the Retina Society Terminology Committee in 1983 [1]. PVR is the most common cause of retinal detachment (RD) repair failure. It is characterized by the growth and contraction of cellular membranes within the vitreous cavity and on both sides of the retinal surface, as well as intraretinal fibrosis, which can lead to tractional RD [2, 3]. The updated Retina Society classification proposed by Machemer et al.[4] in 1991, categorized grade C “posterior PVR” referred to eyes with focal full-thickness retinal folds (C1) or diffuse contraction (C2) located posterior to the equator; “Sub-retinal PVR” referred to eyes with subretinal fibrosis (C3); “Anterior PVR” included eyes with circumferential contraction (C4) or anterior traction on the retina at the vitreous base (C5) alone. Despite substantial advances in vitreoretinal surgical techniques in recent years, including valved trocars and smaller gauge instrumentation, the incidence of PVR has not decreased particularly, remaining around 5–10% [5–8]. Following PVR detachment surgery, the anatomic success rate has been 45–85% [9–14].

Previously published investigations suggested that retinectomy is essential in eyes that have undergone primary pars plana vitrectomy (PPV) for RD as they frequently develop post-operative PVR [10, 15]. In these cases, the careful removal of the retina anterior to the retinotomy is paramount to avoid the development of anterior-loop traction that may lead to tractional ciliary body detachment and hypotony. In addition, the idea of combined management with scleral buckle (SB) has already been raised since it relieves circumferential traction, enhances the tamponade effect of silicone oil (SO), and might be an essential factor in preventing new hole formations after SO removal [16, 17]. The most expected situation is the inferior recurrence of RD with or without a new or reopened retinal break inferiorly in association with SO. A small meniscus of vitreous fluid remains inferiorly when the patient is upright, and the silicone bubble rises slightly superiorly, even with a clinically complete fill of SO. This is because the shape of the internal eye is not precisely rounded like the bubble. The combination of protein, inflammatory, and metaplastic cells and lack of tamponade in this area can lead to further proliferation on the retinal surface in 50–60% of eyes [18, 19], which has been called perisilicone proliferation [20].

This study aimed to investigate surgical and visual outcomes of PPV with inferior retinectomy (extended from 120° to 270°) and a SO exchange to treat recurrent inferior retinal detachment in those previously treated with PPV and SO implantation. The primary outcome was anatomical surgical success, defined as a complete retinal re-attachment at the last follow-up. Secondary outcomes included visual acuity (VA) improvement and postoperative complications.

## Materials and methods

A single-center, retrospective, interventional case series analysis was performed by reviewing the clinical records of 18 patients affected by retinal redetachment due to PVR development inferiorly subsequent to primary PPV and SO implantation for rhegmatogenous RD in follow-up at the *Suel Abujamra Institute, São Paulo, Brazil*. All patients included in the study underwent secondary surgery with large inferior retinectomy and silicone oil filling between 2014 and 2021. Baseline PVR was graded at the last preoperative examination according to the classification proposed by Machemer et al. [4]. The chart review period did not overlap previous investigations on this topic, and no patient in this series was included in any previously published series. This study was conducted in accordance with the tenets of the Declaration of Helsinki, and the reporting of this study conforms to the STROBE statement.

Patients with proliferative diabetic retinopathy, high myopia, penetrating eye injury, uveitis, or more than one previous retinal detachment surgery were excluded from the study. Each patient underwent pre-and postoperative complete ophthalmic examination, which included logarithm of the minimum angle of resolution (logMAR), best-corrected visual acuity (BCVA), applanation tonometry, slit-lamp biomicroscopy, and dilated binocular indirect ophthalmoscopy with scleral depression to evaluate the retinal status and PVR. Dilated binocular indirect ophthalmoscopy, as well as optical coherence tomography were performed to confirm anatomical success after the secondary surgery.

The surgical technique included four-port sclerotomies combined with a 23-gauge chandelier-assisted PPV. SO was first removed using an 18-gauge cannula. Preretinal membranes were removed by peeling with a membrane pick or an intraocular forceps. Full-thickness endocautery was used to delimit the retina before performing vitrector-assisted retinotomy. Retinotomy extension was performed according to the surgeon’s decision at the time of surgery based on retinal shortening (from 120° to 270°), followed by peripheral retinectomy with careful removal of the retina anterior to the incision. Subsequent posterior retinal turnover was performed to remove subretinal membranes, if present, with direct visualization. After flattening the retina with perfluorocarbon liquid, a triple concentric pattern of endolaser was applied to the retinectomy edges. At the end, SO was infused after air-fluid exchange. In all surgeries, 5000 centistokes silicone oil was used as a tamponade (Additional file 1 contains the surgical video S1).

Outcome variables included BCVA, retinal reattachment at final follow-up, and postoperative complications. Values of 0.7 logMAR (equivalent to 20/100 Snellen), 1.3 logMAR (equivalent to 20/400 Snellen), 2.1 logMAR (equivalent to count fingers at 60 cm; or 20/2,000), 2.4 logMAR (equivalent to hand motion at 60 cm; or 20/20,000), and 2.7 logMAR (equivalent to light perception) were assigned [21].

Statistical analyses were performed in SPSS V20, Minitab 16, and Excel Office 2010. The Snellen BCVA measurements were converted into logarithm of minimal angle of resolution units to perform statistical analyses. BCVA (LogMAR) were expressed as mean ± SD. The statistical significance of the BCVA change following surgery was confirmed through the Wilcoxon test. A 95% confidence interval and 5% level of significance were adopted. A p-value of less than 0.05 was considered statistically significant.

## Results

This retrospective study included 18 eyes of 18 patients. The baseline characteristics of the patients are summarized in Table 1. The mean age was 55.3 ± 10.4 (± SD) years (range: 30 to 71 years). All patients were admitted to the *Suel Abujamra Institute (São Paulo, Brazil)* between January 2014 and December 2021 for secondary RD surgery repair. The mean follow-up period was 44.0 ± 31.5 (± SD) months (range: 4 to 96 months). Baseline characteristics of the two groups were homogeneous with no significant statistical difference between groups in terms of initial BCVA, and retina status. All patients were pseudophakic. At the time of surgery, seven patients (38.9%) had previously undergone encircling scleral buckling associated with PPV, with two of them (28.5%) not achieving anatomical success at the last follow-up. At the end of the surgery, it was reported the retina was attached in all cases.

**Table 1:** Demographics and clinical presentation in all patients of the study

Patient number	Initial BCVA (LogMAR)	Final BCVA (LogMAR)	Sex	Age (years)	Follow-up period	PVR grade	Final macular status	Extent of retinectomy (degrees)	Late complications
#1	2.4	2.1	Female	30	4 months	C1	Attached	150^o^	None
#2	2.1	1.3	Male	58	4 months	C2	Attached	180^o^	None
#3	2.4	1.3	Female	68	5 months	C2	Attached	120^o^	None
#4	2.1	2.1	Male	71	46 months	C3	Attached + SB + SO removal	120^o^	None
#5	2.7	2.4	Female	44	45 months	C2	Attached + SB	160^o^	Shallow AC + Optic disk atrophy
#6	2.4	1.3	Male	52	36 months	C1	Detached + SB	270^o^	Re-detachment
#7	2.4	2.4	Male	63	49 months	C2	Detached + SB	120^o^	Re-detachment + Hypotony
#8	2.4	1.3	Male	48	43 months	C3	Attached + SB	180^o^	optic disk atrophy
#9	2.1	1.3	Male	46	44 months	C2	Attached + SB	160^o^	ERM
#10	2.4	2.1	Male	62	27 months	C1	Attached	160^o^	None
#11	2.1	0.7	Male	57	72 months	C3	Attached + SO removal	180^o^	ERM
#12	2.7	1.3	Male	56	84 months	C1	Attached	180^o^	None
#13	1.3	0.7	Female	68	72 months	C1	Attached	180^o^	Cystoid macular edema
#14	2.1	0.7	Male	52	96 months	C3	Attached	180^o^	Cystoid macular edema
#15	1.3	0.7	Male	55	60 months	C1	Attached + SO removal	180^o^	None
#16	2.1	1.3	Female	44	95 months	C1	Attached + SO removal	270^o^	None
#17	2.1	1.3	Male	56	4 months	C2	Attached + SB	180^o^	None
#18	2.1	1.3	Female	65	6 months	C1	Attached	180^o^	None

*BCVA* best-corrected visual acuity, *SB* scleral buckling, *SO* silicone oil, *AC* anterior chamber, *ERM* epiretinal membrane, *PVR* proliferative vitreoretinopathy

**Fig. 1:**
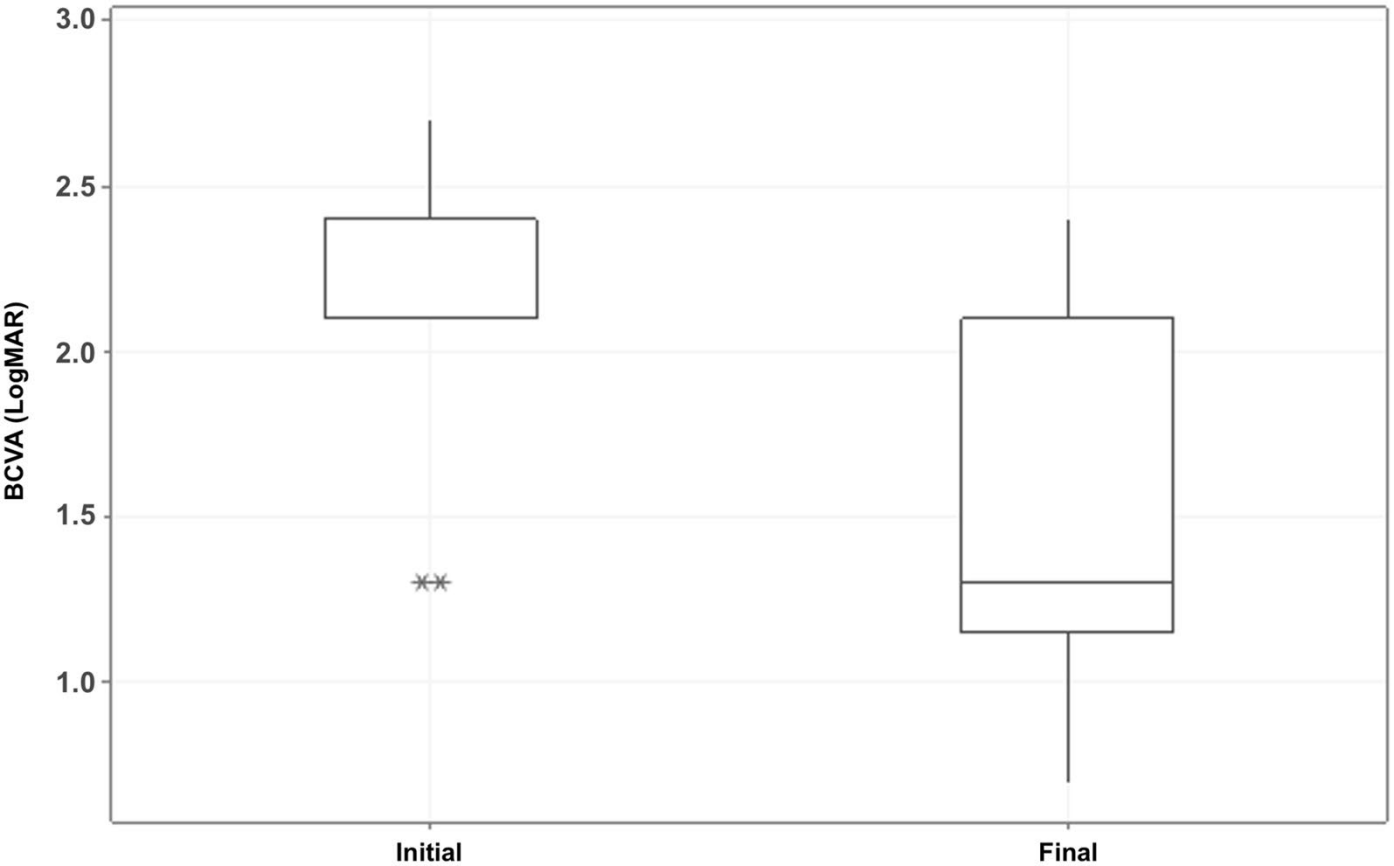
Box-plot graph for BCVA (LogMAR) according to the moment analyzed (initial or final)

Mean preoperative BCVA (LogMAR) improved significantly (P < 0.001) from 2.18 ± 0.38 (median 2.10) to 1.42 ± 0.57 (median 1.30) LogMAR at the last postoperative visit (represented graphically in Fig. 1). BCVA improved in 16 eyes (88.9%), and remained the same in the two (11.1%) eyes that did not achieve anatomical success.

At the last follow-up, an attached retina was reported in 16 patients (88.9%), concluding that there is statistical significance in the final anatomical success (p < 0.001). No intraoperative complications were observed. Four patients (22.2%) underwent an additional procedure with SO removal, remaining with the attached retina until the last follow-up. Postoperative complications included: hypotony (one patient 5.5%), optic disc atrophy (two patients; 11.1%), epiretinal membrane (two patients; 11.1%), retinal re-detachment (two patients; 11.1%), and cystoid macular edema (two patients, 11.1%). Thus, 44.4% of the cases had some type of late postoperative complication compared to 55.6% of patients who did not, but this difference was not statistically significant (p = 0.505).

Table 2 shows the characteristics of the retinal detachment and interventions in all patients in the study. The mean “Interval Time Between RD and Primary Surgery” was 20.6 ± 7.0 weeks; the “Interval Time Between Primary Surgery and Re-RD” was 3.1 ± 0.8 weeks; and the “Interval Time Between Re-RD and Secondary Surgery” was 5.4 ± 1.7 weeks. These three variables have high variability, because the coefficient of variation is greater than 50% (73%, 55%, and 66%, respectively), demonstrating that the data are heterogeneous. In addition, these three variables did not present a statistically significant correlation with final macular status (p-value 0.723, 0.093, and 0.523, respectively), nor with late complications (p-value 0.472, 0.116, and 0.346, respectively).

**Table 2:** Retinal detachment characteristics in all patients of the study

Patient number	Interval time between RD and primary surgery (weeks)	Interval time between primary surgery and re-RD (weeks)	Interval time between re-RD and secondary surgery (weeks)	Presence of PVR prior the primary surgery	Primary intervention	Additional procedure (weeks between secondary surgery and additional procedure)
#1	24	5	4	No	PPV + SO	None
#2	12	2	1	No	PPV + SO	None
#3	1	2	4	No	PPV + SO	None
#4	32	5	6	No	PPV + SB + SO	SO removal (24)
#5	52	6	12	No	PPV + SB + SO	None
#6	22	2	10	Yes	PPV + SB + SO	None
#7	12	1	4	No	PPV + SB + SO	None
#8	32	3	8	No	PPV + SB + SO	None
#9	2	3	1	Yes	PPV + SB + SO	None
#10	32	2	8	Yes	PPV + SO	None
#11	16	6	2	No	PPV + SO	SO removal (24)
#12	16	2	6	No	PPV + SO	None
#13	1	3	12	No	PPV + SO	None
#14	12	6	4	Yes	PPV + SO	None
#15	52	2	1	No	PPV + SO	SO removal (52)
#16	16	3	8	No	PPV + SO	SO removal (36)
#17	12	1	5	No	PPV + SO	None
#18	24	2	2	No	PPV + SO	None

*RD* retinal detachment, *PPV* pars plana vitrectomy, *SB* scleral buckling, *SO* silicone oil, *PVR* proliferative vitreoretinopathy

The correlation of PVR grade with initial and final BCVA, and the extent of the retinectomies, are demonstrated in Table 3. These results were obtained using the Kruskal–Wallis test, and demonstrate that there is no statistical difference between the PVR grade and the results of both the initial and final BCVA, as well as the extent of retinectomy. In addition, the variable “Presence of PVR Prior to Primary Surgery” also had no statistically significant influence on the following variables: BCVA (initial and final), the extent of retinectomy, final macular status, and late complications (p > 0.05).

**Table 3:** Correlation of “PVR grade” with Initial BCVA, final BCVA, and extent of Retinectomy

Z	Mean	Median	SD	N	CI	p-value
Initial BCVA						
C1	2.09	2.25	0.52	8	0.36	0.742
C2	2.30	2.25	0.24	6	0.20	
C3	2.18	2.10	0.15	4	0.15	
Final BCVA						
C1	1.35	1.30	0.53	8	0.37	0.319
C2	1.67	1.30	0.57	6	0.45	
C3	1.20	1.00	0.66	4	0.65	
Extent of retinectomy						
C1	196.3	180.0	46.9	8	32.5	0.182
C2	153.3	160.0	27.3	6	21.9	
C3	165.0	180.0	30.0	4	29.4	

*PVR* proliferative vitreoretinopathy, *BCVA* best-corrected visual acuity, *SD* standard deviation, *CI* confidence interval

The results shown in Table 4 were obtained with the chi-square test, and demonstrate that the PVR grade also does not have a statistical correlation with the final macular status (p-value 0.704), and therefore are variables considered statistically independent.

**Table 4:** Correlation of "PVR grade" with "Final macular status"

	C1		C2		C3		Total	
	N	%	N	%	N	%	N	%
Attached	7	87.5%	5	83.3%	4	100%	16	88.9%
Detached	1	12.5%	1	16.7%	0	0%	2	11.1%
Total	8	44.4%	6	33.3%	4	22.2%	18	100%

p-value = 0.704

Table 5 compared the results obtained in a subgroup with homogeneous characteristics (patient submitted to an additional procedure with SO removal). These results were obtained using the Mann–Whitney test. They showed no statistical difference between patients who did not undergo SO removal and those who experienced this additional procedure, regarding the initial BCVA (p-value 0.054), final BCVA (p-value 0.303), and extent of retinectomy (p-value 0.530). This subgroup (SO removal group) also did not show a statistically significant difference regarding final macular status, when compared to the subgroup that did not undergo an additional procedure (p-value 0.595), and these data are shown in Table 6. Moreover, the primary intervention (PPV + SO, associated or not with SB) did not exert a statistically significant influence on the final macular status (p-value 0.098), as shown in Table 6.

**Table 5:** Comparing “additional procedure (SO removal)” with initial and final BCVA, and the extent of the retinectomy

	Mean	Median	SD	N	CI	p-value
Initial BCVA						
None	2.26	2.4	0.35	14	0.18	0.054
SO Removal	1.90	2.1	0.40	4	0.39	
Final BCVA						
None	1.49	1.3	0.55	14	0.29	0.303
SO Removal	1.20	1.0	0.66	4	0.65	
Extent of retinectomy						
None	171.4	180.0	35.5	14	18.6	0.530
SO Removal	187.5	180.0	61.8	4	60.6	

*SO* silicone oil, *BCVA* best-corrected visual acuity, *SD* standard deviation, *CI* confidence interval

**Table 6:** Correlation of “Final macular status” with qualitative factors

	Attached		Detached		Total		p-value
	N	%	N	%	N	%	
Additional procedure							
None	12	75.0%	2	100.0%	14	77.8%	0.595
SO Removal	4	25.0%	0	0.0%	4	22.2%	
Presence of PVR prior the primary surgery							
No	13	81.3%	1	50.0%	14	77.8%	0.366
Yes	3	18.8%	1	50.0%	4	22.2%	
Primary intervention							
PPV + SB + SO	4	25.0%	2	100.0%	6	33.3%	0.098
PPV + SO	12	75.0%	0	0.0%	12	66.7%	

*SO* silicone oil, *PVR* proliferative vitreoretinopathy, *PPV* pars plana vitrectomy, *SB* scleral buckling

Finally, as shown in Table 7, we used the Spearman correlation to measure the level of correlation between the quantitative factors described. We conclude that there is a statistically significant correlation between "Final BCVA" and "Initial BCVA" (r = 0.654) and between "Final BCVA" and "Extent of Retinectomy" (r = 0.615). Both correlations are classified as "strong". These results mean that the better the initial BCVA, the better the final BCVA. On the other hand, the correlation with "Extent of Retinectomy" shows that the higher the value of "Final BCVA" (proportionally worse BCVA), the lower the value of "Extent of Retinectomy" and vice versa. These data demonstrate that retinectomies with greater extensions had a better final BCVA final, and these values are statistically significant (p-value 0.007).

**Table 7:** Correlation between quantitative factors

	Initial BCVA	Final BCVA	Extent of retinectomy	Interval time between RD and primary Surgery	Interval time between primary surgery and Re-RD
Final BCVA					
Corr (r)	0.654				
p-value	0.003				
Extent of retinectomy					
Corr (r)	0.309	0.615			
p-value	0.212	0.007			
Interval time between RD and primary surgery					
Corr (r)	0.239	0.331	0.010		
p-value	0.340	0.179	0.968		
Interval time between primary surgery and Re-RD					
Corr (r)	0.067	0.123	0.044	0.205	
p-value	0.793	0.628	0.861	0.415	
Interval time between Re-RD and secondary surgery					
Corr (r)	0.388	0.270	0.149	0.205	0.152
p-value	0.111	0.278	0.555	0.414	0.548

*BCVA* best-corrected visual acuity, *RD* retinal detachment, *Corr (r)* correlation

## Discussion

In the current study, we have reported that PPV with large inferior retinectomy and SO tamponade may result in a significant anatomical and functional success rate for retinal re-detachment with inferior PVR cases by relaxing the shortened retina and without alarming late complications. PVR remains the primary cause of unsuccessful RRD surgery, and relaxing retinectomies are mandatory when a complete relief of retinal traction is not possible, even after membrane removal. The cut edge may fibrose and retract back to the posterior pole; however, this situation may still allow good vision as substantiated herein.

PVR is still the major cause for the failure of RRD repair [7]. Given the shortage of pharmacologic alternatives for PVR, the mainstay of treatment for retinal detachments with PVR is surgical intervention, despite the ideal timing of surgery being controversial. Postpone the procedure would allow a greater approach with membrane peel ensuring more effective removal of the membranes [22]. The decision to delay surgery must be deliberated with the macula status and implications on visual prognosis with further postponement. Approximately 77% of postoperative forms of PVR appear within 1 month after retinal detachment surgery, and 95% occur within 45 days [3, 5].

Early re-detachment is defined as detachments that occur within the first 6 postoperative weeks, while late re-detachment is defined as detachments that occur after 6 postoperative weeks. According to the data available in the literature, more than 80% of re-detachments cases occur within the first 6 weeks of primary surgery [23]. In our study, all patients included had a diagnosis of re-detachment within 6 weeks after the primary surgery and were therefore classified as early re-detachments.

Following PVR detachment surgery, the anatomic success rate has been reported to be 45–85% [9, 24–26]. Furthermore, even with anatomic success, patients can have poor visual outcomes [27]. In a previously published study, Mendes et al*.* [15] reported a final reattachment in 94.7% of the patients after repeated surgery attempts. In their study, four patients (10.5%) needed a third surgical procedure, including enlargement of the retinectomy from 270° to 360°. The authors also found a positive association between retinectomy extension and postoperative visual acuity.

In the study herein presented, we explored anatomical and functional outcomes of 18 eyes with inferior retinal redetachment due to PVR, with previous PPV with SO tamponade as primary treatment. All patients underwent PPV with inferior retinectomy (extended from 120° to 270°) and SO exchange (5000 centistokes). The extension of retinectomies reflects not only the severity of the retinal disease but was also performed to obtain a good SO tamponade of the ends of the retinectomy when the patient was in the upright position. At the final follow-up, 88.9% (16 out of 18 eyes) attained complete retinal reattachment. The rate achieved is slightly higher compared to previous studies of PVR detachment surgeries. The fact that all the patients in our study underwent large retinectomies may explain the higher success rate. The BCVA improved after surgery in 16 of our patients (88.9%) and remained stable in the two eyes (11.1%) that did not achieve anatomical success, and therefore there were no cases with worsening of BCVA.

In our study, the main cause of anatomical and functional failure was retinal detachment due to recurrent PVR that developed at the site of retinectomy causing further retinal shortening (two eyes; 11.1%). We also report a single case (5.5%) of postoperative hypotony, in which the patient refused to undergo further surgeries. Postoperative hypotony is a common complication after large retinectomy and previous studies have reported a 15–40% rate in eyes that underwent a 360° retinectomy [26, 28–30].

Our study also allowed us to demonstrate the correlative analysis of different qualitative and quantitative variables between them. The results presented herein showed that the interval time between “RD and primary surgery”, “primary surgery and re-RD”, and “re-RD and secondary surgery” did not present a statistically significant correlation with final macular status nor with late complications. Despite these data, we reinforce that all patients included were classified as early re-detachments (within the first 6 postoperative weeks).

The PVR grade, as well as the presence of PVR prior to primary surgery, did not demonstrate statistically significant relevance when correlated with BCVA (initial and final), the extent of retinectomy, and final macular status, as shown in Tables 3 and 4.

Although the main purpose of the study was to investigate anatomical and functional outcomes referring to a frequent and specific situation (eyes with inferior retinal redetachment due to PVR, with previous PPV with SO tamponade as primary treatment), the data available for statistical analysis allowed us to investigate a homogeneous subgroup characterized by having undergone an additional procedure with SO removal. The results showed no statistical difference between those two subgroups (patients who did not undergo SO removal and those who experienced the additional procedure) regarding the BCVA (initial and final), the extent of retinectomy, and final macular status. The patients who underwent SO removal had a good clinical appearance of the retina, with no evidence of PVR or retinal shortening before the additional procedure. This favorable scenario may justify the anatomical and functional outcomes achieved in this subgroup. However, not all patients had ideal conditions for this additional procedure, evidencing the importance of a careful and individual assessment for the indication of the SO removal.

An important piece of information obtained in our study is that the initial BCVA is positively correlated with the final BCVA (r = 0.654), and this correlation is statistically significant. This data allows us to conclude that patients who present better BCVA when diagnosed with re-RD, have a higher probability of better final BCVA, and this correlation was classified as "strong". Furthermore, the final BCVA is negatively correlated with the extent of the retinectomy (r = 0.615). This correlation demonstrates that retinectomies with greater extensions had a better final BCVA final, and these values are statistically significant (p-value 0.007).

This study has a few limitations that should be mentioned. The small number of patients and the retrospective data collection are the most important limitations. This can be explained by the complexity of the cases and the lack of data. In addition, no direct comparison with a control group was performed, and the surgeries were not performed by a single surgeon. Despite this, the surgeries were performed by experienced retinal surgeons. Nevertheless, the study has strengths, including the long follow-up interval, and the homogeneous subset of retinal detachments and lens status.

This study describes anatomical and functional outcomes of PPV with large inferior retinectomy and SO tamponade for the treatment of re-detachment through inferior PVR in eyes that had previously undergone PPV and SO filled. We evidenced that this procedure may achieve a significant anatomical and functional success rate, besides presenting low post-operative complications index. In addition, patients with better BCVA at the time of re-RD diagnosis, as well as cases of retinectomies with greater extensions, may achieve better functional results.

## Supplementary Information

**Additional file 1: Video S1.** The surgical technique performed in all cases: four-port sclerotomies combined with a 23-gauge chandelier-assisted pars plana vitrectomy; Silicone oil was first removed using an 18-gauge cannula. Retinectomy extension was performed according to the surgeon’s decision at the time of surgery based on retinal shortening (from 120° to 270°). In all surgeries, 5000 centistokes silicone oil was used as a tamponade.

## Data Availability

The authors are responsible for the data in the manuscript and assure full availability of the study material.
